# A high-throughput *de novo *sequencing approach for shotgun proteomics using high-resolution tandem mass spectrometry

**DOI:** 10.1186/1471-2105-11-118

**Published:** 2010-03-05

**Authors:** Chongle Pan, Byung H Park, William H McDonald, Patricia A Carey, Jillian F Banfield, Nathan C VerBerkmoes, Robert L Hettich, Nagiza F Samatova

**Affiliations:** 1 Computer Science and Mathematics Division, Oak Ridge National Laboratory, Oak Ridge, TN, USA; 2 Chemical Sciences Division, Oak Ridge National Laboratory, Oak Ridge, TN, USA; 3 Department of Computer Science, North Caroline State University, Raleigh, NC, USA; 4 Department of Earth and Planetary Science, University of California, Berkeley, CA, USA; 5 Department of Computer Science, University of Tennessee, Knoxville, TN, USA; 6 Current address: Proteomics Laboratory, Mass Spectrometry Research Center, School of Medicine, Vanderbilt University, Nashville, TN, USA

## Abstract

**Background:**

High-resolution tandem mass spectra can now be readily acquired with hybrid instruments, such as LTQ-Orbitrap and LTQ-FT, in high-throughput shotgun proteomics workflows. The improved spectral quality enables more accurate *de novo *sequencing for identification of post-translational modifications and amino acid polymorphisms.

**Results:**

In this study, a new *de novo *sequencing algorithm, called Vonode, has been developed specifically for analysis of such high-resolution tandem mass spectra. To fully exploit the high mass accuracy of these spectra, a unique scoring system is proposed to evaluate sequence tags based primarily on mass accuracy information of fragment ions. Consensus sequence tags were inferred for 11,422 spectra with an average peptide length of 5.5 residues from a total of 40,297 input spectra acquired in a 24-hour proteomics measurement of *Rhodopseudomonas palustris*. The accuracy of inferred consensus sequence tags was 84%. According to our comparison, the performance of Vonode was shown to be superior to the PepNovo v2.0 algorithm, in terms of the number of *de novo *sequenced spectra and the sequencing accuracy.

**Conclusions:**

Here, we improved *de novo *sequencing performance by developing a new algorithm specifically for high-resolution tandem mass spectral data. The Vonode algorithm is freely available for download at http://compbio.ornl.gov/Vonode.

## Background

Tandem mass spectrometry (MS/MS) has become an important method for characterizing complex protein mixtures. Since the emergence of this high-throughput technology, two complementary data analysis approaches have been pursued: a database searching approach and a *de novo *sequencing approach. The former identifies peptide sequences from a protein database by matching their predicted tandem mass spectra to measured tandem mass spectra; whereas the latter infers partial or complete peptide sequences directly from measured tandem mass spectra. Due to its high identification accuracy and the rapid expansion of genomic sequence data, the database searching approach, enabled by popular algorithms such as Sequest [1] and Mascot [2], is routinely used in current proteomics workflows. In contrast, although many *de novo *algorithms have been developed [3-14], they have not been widely used in high-throughput proteomics workflows. A high-throughput *de novo *sequencing capability is critically needed for detection of post-translational modifications (PTMs), characterization of amino acid polymorphisms, and identification of proteins not represented in sequence databases.

*De novo *sequencing has been actively pursued using MS/MS data acquired with ion trap instruments, which are the workhorses of many proteomics workflows. However, the relatively poor mass resolution, mass accuracy, and signal-to-noise ratio of ion trap MS/MS data is a large challenge for *de novo *sequencing. The basic idea of *de novo *sequencing is that, if the mass difference between two mass spectral peaks corresponds to an amino acid mass, the two peaks are likely two adjacent fragment ions flanking a residue of the peptide. Because of the low mass accuracy of ion trap MS/MS data, it is common for two unrelated peaks to have a mass difference reasonably close to an amino acid mass by coincidence. Additionally, in ion trap tandem mass spectra, low-intensity peaks of real fragment ions are often obscured by a large number of noise peaks, again interfering with *de novo *sequencing.

In recent years, new hybrid mass spectrometers, such as LTQ-Orbitrap and LTQ-FT from Thermo Fisher Scientific Inc., have become more common in proteomics workflows [15-17]. These instruments combine the all-around MS/MS capability of ion trap with the excellent mass analysis capability of Orbitrap and FT-ICR. Typical proteomics measurements take high-resolution full scans with Orbitrap or FT-ICR and MS/MS scans with ion trap to achieve accurate parent mass analysis and fast MS/MS acquisition. These hybrid instruments are also capable of acquiring high-resolution tandem mass spectra by performing peptide fragmentation in the front-end ion trap and then transferring fragment ions to the back-end Orbitrap or FT-ICR for mass analysis. Such high-resolution MS/MS data is more amenable to *de novo *sequencing than MS/MS data acquired on conventional ion trap instruments. Several *de novo *sequencing algorithms, including PepNovo [5,18], DirecTag [13], PEAKS [6] and MSNovo [11], accept both ion trap MS/MS data and high-resolution MS/MS data. Most of existing *de novo *sequencing algorithms can be adapted to high-resolution MS/MS data by tightening up mass error tolerance. *De novo *sequencing has also been combined with a UStag approach for PTM identification using FT-Orbitrap MS/MS data [12,19]. All these algorithms should obtain a significant performance boost by using high-resolution MS/MS data.

Here, we report a new *de novo *sequencing algorithm, Vonode (Freely available at http://compbio.ornl.gov/Vonode), to further exploit the potential of high-resolution MS/MS data by using a unique tag scoring function and a novel type of spectrum graphs. The scoring function of Vonode relies more on mass accuracy information than on ion intensity information for scoring sequence tags. Because of the improved dynamic range and sensitivity of high-resolution MS/MS data, fragment ions with lower intensity could just as likely be y and b ions as those with higher intensity. This is contrary to the intuitive assumption that lower-intensity peaks are less significant. A rigorous way for using intensity information is to model theoretical intensities in a spectrum for a sequence tag, which in turn requires building a reliable statistical model with comparable training data [5]. In comparison, mass accuracy information in high-resolution MS/MS can be well defined statistically and is straightforward to use for scoring.

To find sequence tags, many existing *de novo *sequencing algorithms reconstruct spectrum graphs [20] where only adjacent fragment ions of the same ion type are connected. Artifact vertices are used to represent non-existent complementary ions for lone y or b ions such that adjacent fragment ions of different ion types can be connected. Vonode uses a new type of spectrum graph where every observed product ion is transformed to one and only one vertex and four types of edges are used to represent the four possible relationships among adjacent fragment ions. Although this new type of spectrum graphs makes it algorithmically more difficulty to find sequence tags, it allows scoring of sequence tags based only on the ions observed in a mass spectrum.

Vonode was compared to an established *de novo *sequence algorithm, PepNovo v2.0 [5,18], to test whether these new features improves *de novo *sequencing. PepNovo v2.0 was adapted to high-resolution MS/MS data acquired on an LTQ-FT instrument and its performance was shown to be significantly improved [18]. This makes PepNovo v2.0 directly comparable to Vonode. The *de novo *sequencing performance was benchmarked with 40,297 high-resolution tandem mass spectra acquired from a 24-hour shotgun proteomics measurement of a bacterium *Rhodopseudomonas palustris*. Peptide sequences were first identified for this MS/MS dataset with the Sequest-DTASelect toolchain [1,21]. To further reduce the false discovery rate of database searching, peptide identifications were filtered with parent mass accuracy using a new Perl program, called SQAMA (SQt Accurate Mass Annotator). A total of 14,907 spectra were identified with a false discovery rate of 0.09%. These confident peptide identifications were used to verify sequence tags generated by *de novo *sequencing algorithms. From this benchmark dataset, the Vonode algorithm inferred sequence tags for 11,422 spectra at an average length of 5.5 residues using the consensus sequence tag approach. The accuracy of inferred consensus sequence tags was 84%. In comparison, the PepNovo v2.0 algorithm generated sequence tags for 2,573 spectra with an average length of 6.0 residues using a score cutoff of 0.8. The accuracy of top sequence tags from PepNovo v2.0 was 65%. Note that Vonode cannot be compared to a new version of PepNovo, PepNovo+ [22,23], for high-resolution MS/MS data, because so far PepNovo+ has only one model for low-resolution ion trap MS/MS data. Although many other *de novo *sequencing algorithms [4,6,11-14] are available for additional performance benchmarking, the comparison of Vonode to the established and widely-used PepNovo v.2.0 program showed that algorithmic concepts behind Vonode's scoring function and the new type of spectrum graphs are important for *de novo *sequencing to take full advantage of high-resolution MS/MS data.

## Methods

### Algorithm Description

In an MS/MS measurement, a peptide is isolated by its *m/z *and fragmented with collision-induced dissociation. The product ions are then analyzed using an MS^2 ^scan. Most high-abundance product ions are y-ion type or b-ion type, generated from cleavage of peptide bonds. B-ions are fragments on the N-terminal side and numbered as b1, b2 and so on according to the number of residues they contain. Y-ions are fragments on the C-terminal side and similarly numbered as y1, y2 and so on. Other product ions can form directly from the cleavage of other bonds or indirectly from secondary fragmentation (neutral loss) of primary product ions. Besides product ions' peaks, tandem mass spectra are also populated with noise peaks.

Formally, let us define a tandem mass spectrum with a neutral parent ion mass P, a mass array {m_0_, m_1_, m_2_, ..., m_n _in ascending order and a corresponding relative abundance array {a_0_, a_1_, a_2_, ..., a_n_. The mass-abundance pair (m_0_, a_0_) has a mass of zero and a relative abundance of 100%. All other pairs (m_i_, a_i_), 1 ≤ i ≤ n, correspond to neutral monoisotopic masses and relative abundances of measured product ions. Mass spectral peaks are deisotoped and assigned a charge state in the pre-processing step by the Vonode algorithm. Peaks that fail in deisotoping are removed.

The *de novo *sequencing problem is to infer partial sequences of a peptide (sequence tags) from its tandem mass spectrum. A sequence tag is represented as M_p_R_1_R_2_...R_m_M_q_, where R_1_R_2_...R_m _is a sequence of amino acid residues and M_p _and M_q _are two residual masses to the two peptide termini. A residue R_i_, 1 ≤ i ≤ m, belongs to an amino acid set **A **= {A, C, D, E, F, G, H, K, M, N, P, Q, R, S, T, V, W, J, Y}. Let M(Ω) be the mass for the amino acid Ω, Ω ∈ **A**. Note that two isobaric amino acids, I and L, are represented with one letter, J, such that every amino acid in set **A **has a unique mass. Sequence tags are bidirectional in that M_p_R_1_R_2_...R_m_M_q _is equivalent to M_q_R_m_R_m-1_...R_1_M_p_. A sequence tag for a tandem mass spectrum is considered correct, if the sequence tag, in either direction, matches exactly a portion of the peptide sequence.

Given multiple sequence tags inferred for a tandem mass spectrum, a longer sequence tag is more informative and, at the same time, more likely to be incorrect as the result of incorporation of a residue with wrong amino acid assignment. Generally, *de novo *sequencing algorithms attempts to find the longest sequence tag that is correct for a certain probability. The Vonode algorithm finds optimum sequence tags in four steps: (1) spectrum graph construction, (2) sequence graph construction, (3) sequence tag searching, and (4) sequence tag scoring.

#### Step 1: Spectrum graph construction

**Figure 1:**
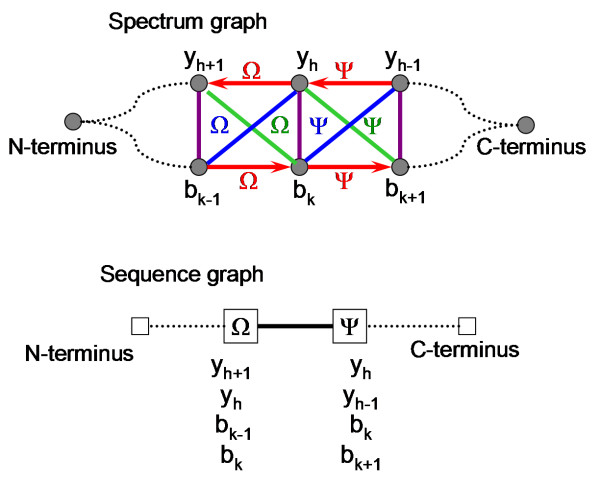
**Model spectrum graph and sequence graph for two adjacent residues Ω and Ψ**. In the spectrum graph, vertices representing y and b ions are connected by four types of edges: arrow edges (Red arrows), forward-slash edges (Blue lines), backslash edges (Green lines), and vertical-bar edges (Purple lines). The spectrum graph is transformed to a sequence graph, where two vertices representing residues Ω and Ψ are connected by a traversable edge (Black solid line).

A spectrum graph is constructed from a tandem mass spectrum, given the amino acid set **A**. A vertex in the spectrum graph, v_i_, represents a product ion (m_i_, a_i_), 0 ≤ i ≤ n. Four types of edges are constructed in a spectrum graph, namely arrow edges, forward-slash edges, backslash edges, and vertical-bar edges. Figure 1 illustrates how edges in the spectrum graph are expected to connect y ions and b ions with one another. Let Ω be an amino acid (Ω ∈ **A**) at the k^th ^residue in a peptide of length (k + h) residues. This residue is flanked by four product ions, y_h_, y_h+1_, b_k-1_, and b_k_. An **arrow edge **(red arrows) is expected to connect a y ion (y_h_) to the next y ion (y_h+1_) or a b ion (b_k-1_) to the next b ion (b_k_). A **forward-slash edge **(blue lines) is expected to connect a y ion (y_h_) with the next complementary b ion (b_k-1_). A **backslash edge **(green lines) is expected to connect a y ion (y_h+1_) with the previous complementary b ion (b_k_). A **vertical-bar edge **(purple lines) is expected to connect two complementary y and b ions (between y_h+1 _and b_k-1 _and between y_h _and b_k_).

**Figure 2:**
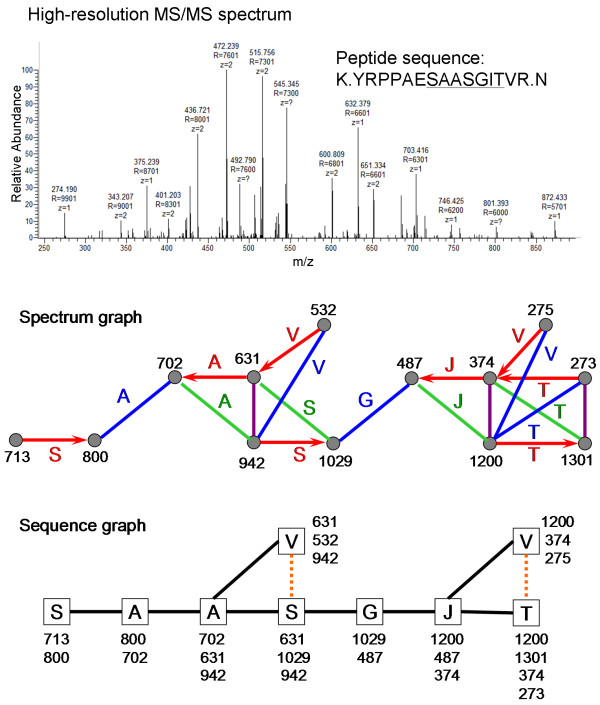
**Spectrum graph and sequence graph for a measured tandem mass spectrum**. Every detected product ion is represented by one and only one vertex in the spectrum graph. Many isolated vertices that are not connected to other vertices are not shown in the figure. The spectrum graph is transformed to a sequence graph for sequence tag searching. The orange dotted lines represent short-circuit edges that prohibit the two connected vertices from being in a sequence path.

Given a set of vertices with unknown identity in a spectrum graph, edges are constructed between two vertices using their masses. Let ε be the relative mass error for an edge, which is the difference between the measured mass combination of two vertices and the expected mass combination given amino acid masses (M(Ω)) and the parent mass (P). Let Δ be the relative mass error tolerance (maximum relative mass error allowed). Two vertices, v_i _and v_j_, with respective masses of m_i _and m_j_, 1 ≤ i < j ≤ n and m_i _< m_j_, are connected by: (i) an arrow edge, if | (m_j _- m_i_) - M(Ω) | = ε < Δ; (ii) a vertical-bar edge, if | (m_j _+ m_i_) - P | = ε < Δ; (iii) a forward-slash edge, if | P - (m_j _+ m_i_) - M(Ω) | = ε < Δ; and (iv) a backslash edge, if | (m_j _+ m_i_) - P - M(Ω) | = ε < Δ. The relative mass errors of spectrum edges are generally smaller than the mass errors of ions, because ion in a spectrum usually have mass errors in the same direction from out of mass calibration and the mass errors can cancel part of each other out in the relative mass error calculation. Figure 2 shows the spectrum graph constructed from a tandem mass spectrum of +3 peptide K.YRPPAESAASGITVR.N. The y and b ions of this peptide could provide information for a sequence tag of SAASGJT. Two vertices that represent two adjacent y or b ions measured with sufficient mass accuracy should be connected by an edge of appropriate type. However, two vertices that are not related can also be connected from coincidence of their mass combination. Every edge is associated with a relative mass error ε and an edge type.

#### Step 2: Sequence graph construction

A sequence graph is constructed from a spectrum graph to facilitate sequence tag searching. For clarity, vertices and edges in a sequence graph are referred to as sequence vertices and sequence edges respectively, whereas vertices and edges in a spectrum graph are referred to as spectrum vertices and spectrum edges respectively.

A sequence vertex, representing an amino acid residue, is constructed from interconnected spectrum vertices that correspond to product ions on the two sides of the residue. These spectrum vertices should be interconnected by spectrum edges all labeled with this residue or vertical-bar spectrum edges. In Figure 1, sequence vertex Ω is constructed from spectrum vertices y_h+1_, y_h_, b_k-1_, and b_k _and sequence vertex Ψ is constructed from spectrum vertices y_h-1_, y_h_, b_k+1_, and b_k_. Note that a spectrum vertex can be used for multiple sequence vertices. A sequence vertex for a residue can be composed of two to four spectrum vertices with at least one spectrum vertex from each side of the residue and a sequence vertex should include as many appropriate spectrum vertices as possible. The sequence graph in Figure 2 contains sequence vertices that are constructed from 2, 3 or 4 spectrum vertices. Sequence vertices are labeled with the m/z values of the included spectrum vertices.

Any two sequence vertices that share one or more spectrum vertices are connected by a sequence edge. There are two types of sequence edges, traversable edges and short-circuit edges. A traversable sequence edge (shown as black solid edges in a sequence graph) connects two sequence vertices whose spectrum vertices can be fit into the interconnection pattern shown in Figure 1, allowing for missing spectrum vertices and spectrum edges. A sequence edge is a short-circuit edge (shown as orange dotted edges in a sequence graph), if it cannot be a traversable edge. Figure 2 illustrates construction of sequence edges in a sequence graph. A traversable sequence edge is used to connect two sequence vertices whose residues can be placed next to each other in a sequence tag. A short-circuit edge is used to connect two vertices whose residues cannot be placed in a sequence tag regardless of whether they are next to each other or separated by other residues.

#### Step 3: Sequence tag searching

Potential sequence tags for a tandem mass spectrum are identified by searching for paths in the sequence graph. A path in a sequence graph is defined as a sequence of vertices connected by traversable edges. A valid path is required to have no repeated vertex or traversable edge (the simple path rule) and no short-circuit edge between any two vertices (the short circuit rule). In the sequence graph of Figure 2, the SAASGJT path and the SAASGJV path are the two longest valid sequence paths. The short circuit rule ensures that a path in a sequence graph corresponds to a legitimate interconnection in the spectrum graph for a sequence tag. An example of invalid paths is the VJT path in the sequence graph of Figure 2. This path, corresponding to an invalid interconnection in the spectrum graph, is ruled out by the short circuit edge between vertices V and T in the sequence graph.

There are many possible paths in a non-trivial sequence graph. The constraint of short-circuit edges for valid paths may complicate using the dynamic programming algorithm described previously for *de novo *sequencing [3,5,24]. To identify optimum paths, the Vonode algorithm uses a brute-force enumeration approach that simply finds all possible paths for subsequent evaluation with a scoring function. Compared to an analytical approach for finding optimum paths, the enumeration approach gives the flexibility to use virtually any scoring function and allows identification of not only the optimum path with the highest score, but also other top paths that rank below the optimum path. Path enumeration is efficient for sequence graphs because of the limited number of vertices and a low degree of connectivity in sequence graphs. All possible paths in a sequence graph are found by iterating through every pair of vertices and identifying all possible paths between them. Let ***v ***and ***d ***be two vertices. **FindPath **(***v***, ***d***, **V**, **P**) is a recursive function that finds all possible paths between ***v ***and ***d ***and returns them in a path list **P**. **V **is a temporary vertex list for storing vertices to be used to construct a path. **P **and **V **are passed by reference between recursive function calls to save previous calculation results. Below is the pseudo-code for **FindPath**:

**FindPath **(***v***, ***d***, **V**, **P**)

1.    **If **any two vertices in **V **are connected by a short-circuit edge

2.       **Return**

3.    Push ***v ***onto the back of **V**

4.    **If *v ***and ***d ***are different vertices

5.       **For **every vertex, ***v'***, that is connected to ***v ***by a traversable edge and is not in **V**

6.          **FindPath**(***v'***, ***d***, **V**, **P**)

7.    **Else**

8.       Construct a path from **V **and save the path into **P**

9.    Pop ***v ***out of the back of **V**

10.    **Return**

All legitimate paths between a source vertex ***s ***and a destination vertex ***d ***are found with the functional call **FindPath(*s*, *d*, *ϕ*, *ϕ*)**, i.e., V and P are empty lists (*ϕ*) in the beginning. Some sequence graphs can be divided into connected components. A connected component is defined as a maximal subgraph connected by traversable edges. For these sequence graphs, sequence paths from each component are found and evaluated independently of paths in other components.

#### Step 4: Sequence tag scoring

Top sequence tags for a tandem mass spectrum are identified by scoring and ranking all valid paths in the sequence graph. The score function used by the Vonode algorithm is based on mass accuracy information of product ions measured in the tandem mass spectrum. In the step of spectrum graph construction, every spectrum edge is assigned with a relative mass error, which is required to be less than the error tolerance (Δ). The relative mass error is standardized to an edge weight by the function, ω = 2(1 - **pnorm**(ε, 0, Δ/2)), where ε represents the relative mass error variable and ω represents the edge weight variable. The function, **pnorm**(ε, 0, Δ/2), calculates the lower-tail cumulative probability for the variable ε from a normal distribution function with a mean of 0 and a standard deviation of Δ/2. Because the relative mass error has a range of [0, Δ], the standardization assigns every spectrum edge a weight between 0.05 and 1.00. The larger the relative mass error of a spectrum edge is, the less weight the spectrum edge has.

A sequence path is scored by enumerating all spectrum edges that are covered by the sequence path. The score of a sequence path is simply the sum of the weights of all covered spectrum edges. The top-scoring sequence paths in a sequence graph are those that cover many spectrum edges with low relative mass errors. The top three sequence paths in Figure 2 are SAASGJT (score = 12.8), AASGJT (score = 12.2), and ASGJT (score = 11.6).

Given a large set of tandem mass spectra, the Vonode algorithm attempts *de novo *sequencing for all tandem mass spectra and then filters tandem mass spectra based on the score of the top sequence tag. The default score cutoff is 4.0.

### Proteome sample preparation

*R. palustris *CGA010 strain was grown in defined growth medium to mid-log phase. Cells were harvested and lysed by sonication in ice-cold wash buffer. Cell lysates were then treated with 6 M guanidine and 10 mM dithiothreitol (DTT) (Sigma Chemical Co. St. Louis, MO) at 60°C for 1 hour for protein denaturation and disulfide bond reduction. After six-fold dilution with 50 mM Tris-HCl/10 mM CaCl_2 _(pH 7.8), the proteome sample was digested at 37°C with sequencing grade trypsin (Promega, Madison, WI). The sample was finally reduced with 20 mM DTT for 1 hour at 60°C and desalted using C18 solid-phase extraction (Sep-Pak Plus, Waters, Milford, MA).

### LC-MS/MS analysis

The processed proteome sample was examined with a 24-hour two-dimensional liquid chromatography-tandem mass spectrometry analysis (2D LC-MS/MS) as described previously [25-27]. Briefly, the sample was first separated by twelve-step strong cation ion exchange liquid chromatography and then by continuous gradient reverse phase liquid chromatography. Eluted peptides were electrosprayed at 3.6-kV distal electrospray voltage into an LTQ-Orbitrap mass spectrometer (Thermo Fisher Scientific, San Jose, CA). Tandem mass spectrometry analysis was performed with each full scan (400-1700 m/z) followed by five data-dependent MS/MS scans at 35% normalized collision energy. Dynamic exclusion was enabled. The full scans were acquired with 2-microscan averaging at resolution 30,000, AGC target 500,000, and maximum ion injection time 500 ms. The MS/MS scans were acquired with 2-microscan averaging at resolution 7,500, AGC target 200,000, and maximum ion time 1,000 ms.

### MS/MS data extraction

The proprietary Finnigan RAW files were converted to FT1 files and FT2 files with a Visual Basic program called RAW2FT2. The RAW2FT2 program requires Finnigan XDK 2.0 to run. The FT1 and FT2 file formats are simple extensions of the MS1 and MS2 file formats [28], respectively, for high-resolution data. In MS1 and MS2 files, every mass spectrum is represented with two columns of data for m/z and intensity. In FT1 and FT2 files, every mass spectrum is represented with seven columns of data in the order of m/z, intensity, resolution, background, noise, and charge state, all of which are extracted from RAW files. All tandem mass spectra have header information for monoisotopic parent mass and charge state in FT2 files. MS/MS data from other instrument venders need to be converted to the FT1 and FT2 formats to be analyzed by Vonode.

### Accurate parent mass filtering of Sequest identification

All MS/MS scans were searched with the Sequest program [1] against a concatenated forward and reverse *R. palustris *protein database with common protein contaminants (Peptide mass tolerance 3 Da, fragment ion tolerance 0.5 Da, trypsin cleavage with up to 4 internal cleavage sites) [29]. Search results were reformatted from OUT files to SQT files [28]. Parent mass accuracy of all peptide identifications was calculated with the SQAMA program in three steps. First, theoretical isotopic distribution of a peptide is calculated using its amino acid sequence and charge state. Then, observed isotopic distributions are found within ± 0.05-Da m/z windows of the expected isotopic peaks in the 6 full scans surrounding the MS/MS scan. Every isotopic peak in observed isotopic distributions represents an independent mass measurement. Finally, the mass error for a peptide identification is calculated as an average of all observed isotopic peaks' mass errors weighted by their intensities. Mass errors are reported in the original CalcM+H+ field in the SQT files.

The DTASelect program [21] was used to filter peptide identifications and to assemble peptides into proteins using the following parameters: retaining the duplicate MS/MS spectra for each peptide sequence (DTASelect option: -t 0), fully tryptic peptides only, with a delCN of at least 0.08 and cross-correlation scores (Xcorrs) of at least 1.8 (+1), 2.5 (+2), and 3.5 (+3), and a minimum of two identified peptides for a protein. Identified peptides were further filtered by their parent mass errors calculated by SQAMA. Parent mass errors were normalized by shifting the median of the mass error distribution to zero for systematic mass error correction. Only peptide identifications with a normalized parent mass error less than 0.02 Da were retained as confident peptide identifications. False discovery rates of peptide identification were calculated using the number of peptide identifications from reverse protein sequences.

### Performance benchmarking

The FT2 files of the benchmark dataset were input into the Vonode program for *de novo *sequencing with the following parameters: Relative mass error tolerance (Δ, as defined in the ALGORITHM DESCRIPTION section) = 0.01 Da; Minimum sequence tag length = 3 (To require at least 3 residues in a sequence tag).

For performance comparison, the benchmark dataset was also analyzed with the PepNovo v2.0 program. MS/MS scans in the FT2 files were formatted into individual DTA files. The default DTA file format for Sequest has m/z values with only one digit after the decimal point. The DTA files generated for PepNovo v2.0 from FT2 files have m/z values with five digits after the decimal point to represent the high-resolution data and a measured parent ion charge state. PepNovo was executed with the default Orbitrap tryptic-peptide model (LTQ_ORBI_TRYP). PepNovo reports top 20 sequence tags for every MS/MS scan. The MS/MS scans were filtered by the "Prob" score of the top sequence tag.

Sequence tags inferred by Vonode and PepNovo for a spectrum were verified with the corresponding Sequest identification. All data processing was completed on a Dell Precision 300 workstation with an Intel Core 2 Duo CPU and 2 GB RAM.

## Results and Discussion

### Orbitrap MS/MS measurement and benchmark dataset preparation

**Figure 3:**
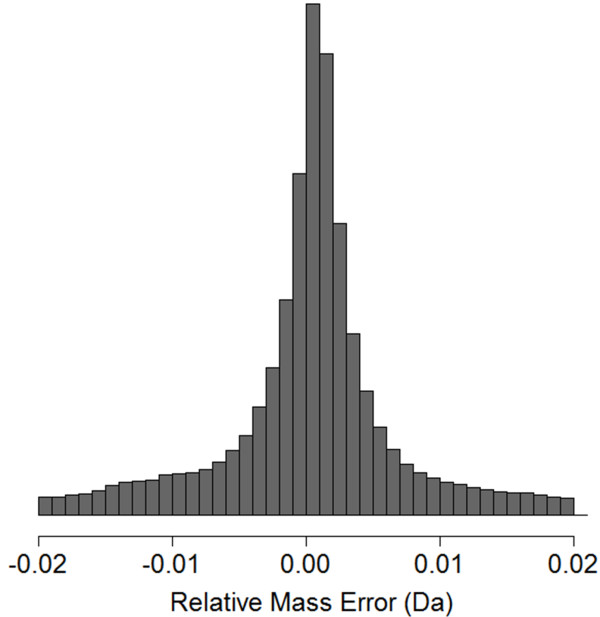
**Distribution of relative mass errors in the Orbitrap MS/MS benchmark dataset**. The relative mass error of a spectrum edge is the mass error of a fragment ion relative to another fragment ion given their expected relationship represented by the spectrum edge. The majority of relative mass errors are distributed between **-**0.01 Da and 0.01 Da in the benchmark dataset.

An *R. palustris *proteome was measured by 2D LC-MS/MS on an LTQ-Orbitrap instrument in the Orbitrap MS/MS mode. The 24-hour analysis yielded 40,297 high-resolution MS/MS spectra. At the configuration of resolution 7500 and 2 microscan averaging, the scanning speed of Orbitrap MS/MS was approximately 50 scans per minute. As a comparison, the scanning speed of LTQ MS/MS (2 microscan averaging) is about 100 scans per minute. The 2-fold decrease in scanning speed of Orbitrap MS/MS reduced the number of MS/MS spectra acquired in the 24-hour 2D LC-MS/MS analysis. Figure 3 shows the distribution of relative mass errors between adjacent fragment ions in tandem mass spectra from the Orbitrap MS/MS benchmark dataset. For comparison, a distribution of relative mass errors from an LTQ MS/MS dataset was shown in Additional File 1, Figure S1. This indicates that Orbitrap MS/MS achieves much higher mass accuracy at the expense of a lower scanning speed.

To benchmark *de novo *sequencing accuracy, peptide identifications were first obtained from this *R. palustris *dataset using database searching and parent mass accuracy filtering. Concatenated forward and reverse database was used to measure false discovery rate of peptide identification [30]. Sequest identifications were assigned to 15,308 tandem mass spectra using default DTASelect filtering criteria yielding a false discovery rate of 0.21%. Further filtering with parent mass accuracy using the SQAMA program yielded identifications for 14,907 spectra with a false discovery rate of 0.09%. In this study, we consider these identifications as gold standard and use them to verify sequence tags inferred by *de novo *sequencing algorithms.

### *De novo *sequencing with the Vonode algorithm

All 40,297 MS/MS spectra were analyzed by the Vonode algorithm in less than 15 minutes. The *de novo *sequencing results were evaluated using the top sequence tag of a spectrum. 14,264 sequenced spectra passed the score threshold of 4.0, yielding sequence tags with an average length of 5.5 residues (Additional File 2, Table S1). For illustration, the *de novo *sequencing and verification results for spectra 1616 - 1644 in cycle 8 are shown in Table 1. 9 spectra in this scan range have a sequence tag assignment, which consists of a sequence tag and two residual masses from the tag to the two termini of the peptide. The two directions of a sequence tag (from left to right or from right to left) are equivalent. Isobaric amino acids L and I are not distinguished in *de novo *sequencing and they are represented by one letter, J, for convenience. Eight of these spectra have Sequest identifications, which were matched to their sequence tags. The sequence tag of a spectrum is verified by Sequest to be correct if the sequence tag in one of the two directions matches exactly to the Sequest peptide sequence. This criterion of verifying a spectrum's sequence tag assignment is more stringent than some of those used in previous studies [5,6,9,14,18,24] in that a spectrum can provide only one sequence tag (the top sequence tag) for verification and only an exact match of all residues in a sequence tag to the corresponding peptide sequence can be considered as correct.

**Table 1:** Sample results of *de novo *sequencing and verification.

Scan #	*De novo *Sequencing				Verification	
	Residual Mass	Tag Sequence*	Residual Mass	Score	Tag Validity	Sequest Identification*
1616	1001.540	APAJG	146.112	8.7	Correct	K.RVFNVLTGDAPAIGK.V
1629	1069.550	VJCJQPK	44.049	5.3	N/A	N/A
1630	897.447	QEKEVAAVJ	128.105	14.8	Incorrect	K.KLVAAVEKEGAGFDLGAYR.D
1635	980.533	EJVQ	146.109	5.3	Incorrect	K.MIHFVPRDNIVQK.A
1636	358.233	DGJMVJA	137.071	22.1	Correct	R.HALVMLGDALR.H
1640	1219.610	TSSMG	589.296	7.6	Correct	R.NYAQLGMSSTPFYQSHGVASK.S
1641	830.437	TJFGA	146.113	11.5	Correct	K.IFTTRPDTLFGAK.F
1643	888.524	AGDGAK	147.079	10.4	Correct	R.FKAGDGAIVNGIAFR.S
1644	128.096	JFDVJA	271.174	19.3	Correct	K.KLFDVLAPR.Y

* Matched sections between the sequence tags and the Sequest identifications are underlined.

* Letter J represents amino acids L or I.

**Figure 4:**
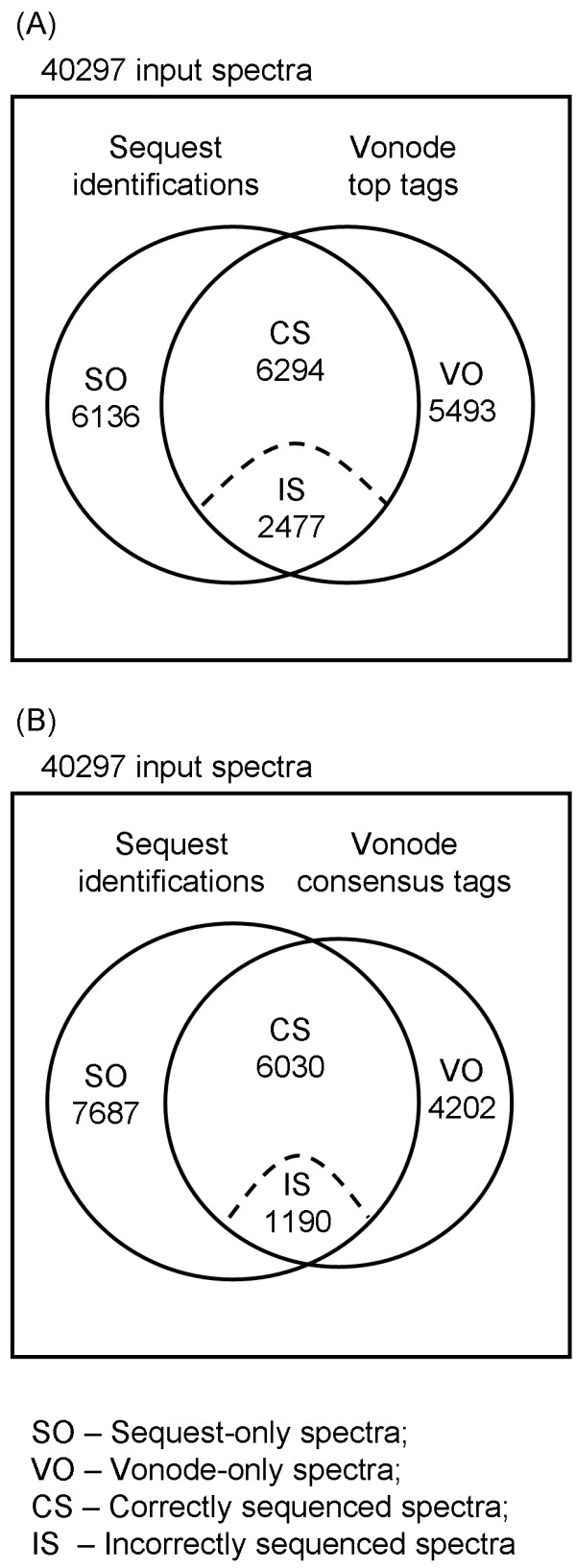
**Overview of the *de novo *sequencing and verification results**. Spectra are categorized into Sequest-only (SO) spectral set, Vonode-only (VO) spectral set, and the intersection set. The intersection set is further divided into correctly sequenced (CS) spectral set and incorrectly sequenced (IS) spectral set. Each spectral set is labeled with the number of spectra it contains. (A) Venn diagram for the top sequence tag results. (B) Venn diagram for the consensus sequence tag results.

A global view of the *de novo *sequencing and verification results is shown with a Venn diagram in Figure 4A. The universal set of all 40,297 spectra is represented by the outer rectangle. The set of 14,907 spectra that have a confident Sequest identification is represented by the circle in the left side. The set of 14,264 spectra that have the top sequence tag assignment by Vonode is represented by the circle in the right side. Note that the Sequest set and the Vonode set do not completely overlap. There is a set of 6,136 Sequest-only spectra that have a Sequest identification but no Vonode sequence tag assignment. *De novo *sequencing requires measurement of fragmentation at more than 4 consecutive peptide bonds for inferring a sequence tag with a minimum length of 3 residues. While fragmentation in these spectra could be informative for Sequest identification, it may not be contiguous for *de novo *sequencing with Vonode.

The intersection of the Sequest set and the Vonode set represents the 8,771 spectra that have both a Sequest identification and a sequence tag assignment. Eight spectra listed in Table 1 belong to this category. As shown in Table 1, this set of spectra can be further divided into two categories - correctly sequenced spectra and incorrectly sequenced spectra - based on the verification result. The accuracy of *de novo *sequencing is defined as the percentage of the correctly sequenced spectra in the intersection set. The accuracy of the top sequence tag assignment is 72% with 6,294 correctly sequenced spectra and 2,477 incorrectly sequenced spectra. Common types of errors in the incorrect sequence tags are described in the last section.

Finally, there is a set of 5,493 Vonode-only spectra that have a sequence tag assignment but no Sequest identification. If we assume that the *de novo *sequencing accuracy calculated from the intersection spectra set remains approximately the same for the Vonode-only spectra set, the majority of these sequence tags would provide correct partial sequence information for many unidentified peptides in the sample. These unidentified peptides could be peptides with non-specific cleavages, peptides whose sequences are not in the protein sequence database (amino acid polymorphisms or novel proteins) or peptides with chemical modifications (biological post-translational modifications or artifact modifications from sample preparation). The potential for mining out biological information from these unidentified peptides is precisely the motivation for developing the *de novo *sequencing approach that complements the database searching approach.

### Algorithm performance comparison

**Figure 5:**
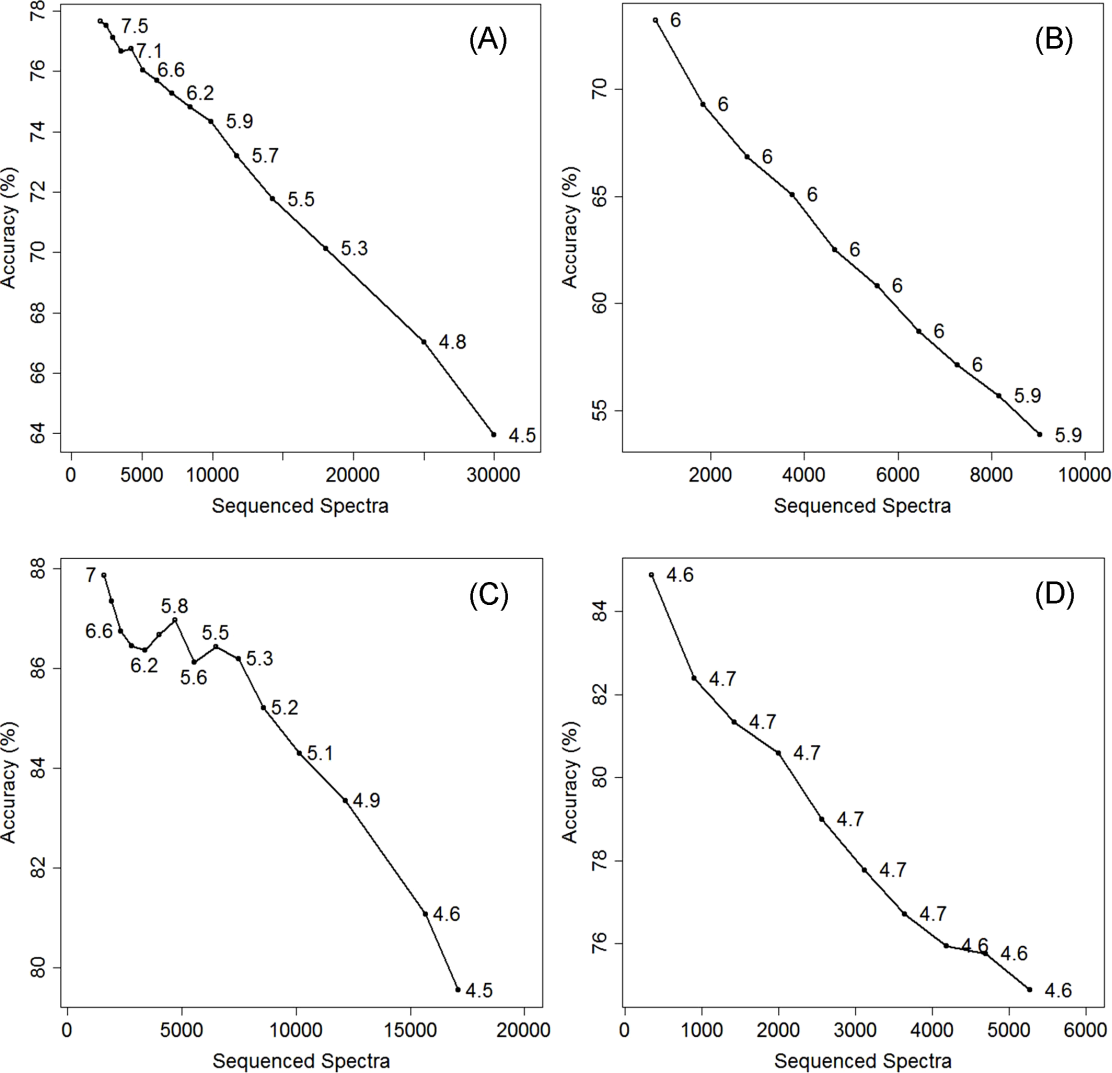
***De novo *sequencing performance comparison of Vonode and PepNovo using varying score thresholds**. The performance is analyzed for Vonode top sequence tags (A), PepNovo top sequence tags (B), Vonode consensus sequence tags (C), and PepNovo consensus sequence tags (D). The thresholds are 1, 2, ..., 15 for Vonode and 0.50, 0.55, ..., 1.00 for PepNovo. The performance at each threshold is defined by the number of sequence spectra (x-axis), the accuracy (y-axis), and the average tag length (text labels of the data points).

In this study, three figures of merit were used to evaluate the performance of the *de novo *sequencing algorithms. The first figure of merit is the accuracy as defined above. It is calculated from the intersection spectra as a simple estimate of the probability for a sequence tag assignment to be correct. The second figure of merit is the average length of sequence tags inferred from a benchmark dataset. A longer sequence tag is more informative and also more likely to be incorrect. Given the same accuracy, an algorithm that has larger average tag length is considered to be better. The third figure of merit is the number of spectra that an algorithm can assign a sequence tag to in a benchmark dataset. A larger percentage of sequenced spectra from a shotgun proteomics measurement can provide higher proteome coverage and higher average protein sequence coverage. The *de novo *sequencing performance of Vonode was evaluated using the three figures of merit at varying score thresholds and a constant minimum tag length threshold of three residues (Figure 5A). The figures of merit for the top sequence tag result filtered with the score thresholds of 4.0 and 8.0 are also shown in Table 2. By increasing the score threshold for filtering sequence tags, the accuracy and the average tag length were improved at the expense of greatly reduced number of *de novo *sequenced spectra.

**Table 2:** Comparison of *de novo *sequencing performance.

Algorithm	Threshold	Sequence Tag	Intersection Spectra	Accuracy	Average Tag Length	Sequenced Spectra
Vonode	8.0	Top	4897	75%	6.2	7142
	4.0	Top	8771	72%	5.5	14264
	4.0	Consensus	7220	84%	5.0	11422
PepNovo	0.9	Top	1293	70%	6.0	1821
	0.8	Top	2460	65%	6.0	3716
	0.8	Consensus	2160	78%	4.7	3253

The performance of Vonode was compared to the PepNovo v2.0 algorithm using the three figures of merits at varying score thresholds (Figure 5). The PepNovo performance using thresholds of 0.8 and 0.9 are also shown in Table 2. The Vonode results filtered at threshold 8.0 have approximately the same average tag length as the PepNovo results. The accuracy of Vonode is better than PepNovo with either cutoff. More importantly, Vonode generated a much larger number of sequence tags than PepNovo from the same benchmark dataset. The performance of Vonode and PepNovo was also compared at different charge states. Spectra were grouped by parent ion charge states (Z = +1, Z = +2, and Z ≥ +3) and the performance was evaluated for each charge state group separately. Vonode performed equally well for charge state +2 and charge states +3 and higher, but much worse for charge state +1 (Additional File 3, Figure S2, Part A). PepNovo performed much better for charge state +2 than other charge states (Additional File 3, Figure S2, Part B). Vonode and PepNovo had a similar performance for charge state +2, but Vonode outperformed PepNovo in the other two charge state groups. The *de novo *sequencing performance of Vonode and PepNovo was also compared at different ranges of mass coverage. Mass coverage is defined for a spectrum as the percentage of the parent peptide mass explained by a sequence tag. Mass coverage was designed to normalize the sequence tag size using the parent peptide size. Performance of Vonode and PepNovo was compared in three mass coverage ranges: 0% ~20%, 20% ~40%, and 40% ~100% (Additional File 4, Figure S3). In the 0% ~20% range, Vonode produced shorter sequence tags at higher accuracy from more spectra than PepNovo. In the other two mass coverage ranges, Vonode sequenced more spectra than PepNovo at comparable average tag length and accuracy.

The superior performance of Vonode is attributed to two key algorithmic innovations. First, a new type of spectrum graph was developed for sequence tag evaluation. Spectrum graphs were first proposed by Bartels *et al *[20] and have been used in many *de novo *sequencing algorithms. In the Bartels type of spectrum graphs, only one type of edge is used, which connects adjacent fragment ions of the same ion type. Artifact vertices are created to represent absent complementary ions for a lone y or b ion to capture the relationships between adjacent fragment ions of different ion types via those artifact vertices. In this study, a new type of spectrum graph was developed to use four types of edges to represent the four possible relationships among adjacent fragment ions (Figures 1 and 2), avoiding creation of any artifact vertices. Every observed product ion is transformed to one and only one vertex in spectrum graph. The score for a sequence tag is the total weight of all spectrum edges covered by the sequence tag. This scoring function rewards a fragmentation site defined by a pair of complementary y and b ions much more than one defined by a lone y or b ion. A residue derived from two adjacent pairs of complementary y and b ions is rewarded with the total weight of six spectrum edges; whereas a residue derived from two adjacent lone fragment ions is rewarded with the weight of only one spectrum edge (Figures 1 and 2).

Second, the weight of a spectrum edge is calculated based on its relative mass error without using intensity information. False edges connecting two unrelated ions were expected to be more likely to have a high relative mass error than true edges connecting two y or b ions. Based on the distribution of relative mass errors in the benchmark dataset (Figure 3), the relative mass error of a spectrum edge was standardized to a weight between 0 and 1. Higher edge weights correspond to lower relative mass errors. This simplistic scoring function of weighing spectrum edges with relative mass errors was designed to take advantage of the improved mass accuracy, sensitivity, and dynamic range of Orbitrap and FT-ICR mass analyzers.

### Development of consensus sequence tag approach

If the top sequence tag of a spectrum is incorrect, the sequence tag with the second highest score for this spectrum could be correct. One can use the top two sequence tags of a spectrum to match its Sequest identification and consider this spectrum to be correctly sequenced if one of the top two sequence tags matches the Sequest identification. The accuracy of the top sequence tags at threshold 4.0 was 72%. By adding the second best sequence tag of every spectrum into consideration, the accuracy was improved to 82%. Adding the third best tag increased the accuracy further to 86%. There was a diminishing return on the accuracy improvement by including more lower-ranking sequence tags. Considering multiple top ranking sequence tags for a spectrum conveniently improved the accuracy, but it would also complicate the subsequent step of using sequence tags for sequence polymorphism characterization and chemical modification identification. A consensus sequence tag approach was proposed to obtain the significant accuracy gain of including the second best sequence tag without reporting two sequence tags for a spectrum. A consensus sequence tag is simply the maximum common sub-sequence between the top two sequence tags in a spectrum. It is guaranteed to be correct if at least one of the top two sequence tags is correct.

The Vonode *de novo *sequencing results were analyzed with the consensus sequence tag approach at varying score thresholds (Figure 5C). Consensus sequence tags were obtained from 11422 spectra using a score threshold of 4.0 and a minimum tag length of three residues (Figure 4B and Additional File 5, Table S2). Figures of merit of this consensus sequence tags approach are shown in Table 2. The consensus tag of a spectrum is at least one residue shorter than the top sequence tag. A spectrum with a consensus sequence tag shorter than 3 residues was discarded. Consensus sequence tags were also extracted from the PepNovo results using varying score thresholds (Figure 5D). The performance of the consensus sequence tag approach was compared to the top sequence tag approach for both Vonode and PepNovo at different charge states and different mass coverage ranges (Additional Files 3, Figure S2, and Additional File 4, Figure S3). Compared to top sequence tags, consensus sequence tags have a greatly improved accuracy at the expense of reduced total number of sequenced spectra and lower average tag length (Table 2). Top sequence tags from Vonode and PepNovo are generally extensions of lower scoring tags or differ from lower scoring tags only in the end residues (Additional File 2, Table S1) and taking consensus sequence tags has the effect of trimming off unreliable residues from the ends of top sequence tags. We think that the consensus sequence tag approach is a better balance among the three figures of merit (the accuracy, the total number of sequenced spectra, and the average tag length) than the top sequence tag approach.

### Characterization of *de novo *sequencing errors

1190 consensus sequence tags and 2477 top sequence tags failed to match Sequest identifications in the incorrectly sequenced spectral set (Figure 4). The majority of these sequence tags are different from the corresponding Sequest identifications by one or two residues. The errors in those incorrect sequence tags are not random amino acid substitutions from their Sequest identifications. There were substitution errors that occur rarely, such as the substitution between two nearly isobaric amino acids, Q and K. The masses of amino acids Q and K are different by only 0.036 Da. With ion trap MS/MS data, it is almost impossible to resolve such a small mass difference to distinguish these two amino acids. In this Orbitrap MS/MS dataset, there were only 0.3% of incorrect sequence tags (3 consensus sequence tags and 7 top sequence tags) that had the substitution error between amino acids Q and K.

Five common types of minor errors by Vonode were characterized (Table 3). An incorrect sequence tag is categorized into one of the five error types if it can be corrected by making a single sequence change of that type. Many wrong sequence tags could be corrected by substituting an amino acid Q with two amino acids G and A (Table 3). An example for this error type is the incorrect sequence tag for spectrum 1630 in Table 1. The combined mass of G and A is exactly the same as the mass of Q. When the fragment ions between G and A are missing in a spectrum, the residue doublets, GA or AG, would be mistakenly replaced by a single residue of Q in the sequence tag. In this benchmark dataset, a residue Q in a sequence tag has an approximately 12% probability to actually be GA or AG. This probability of the Q assignment ambiguity should be taken into account when using a sequence tag containing Q. For the same reason, the residue doublet, GG, can be mistaken for a single residue of N. Together, these two types of errors account for 18% incorrect consensus sequence tags and 12% incorrect top sequence tags.

**Table 3:** Characterization of common *de novo *sequencing errors.

*De novo *sequencing error types	Top sequence tags		Consensus sequence tags	
	Counts	Percentage	Counts	Percentage
Substitution of a Q by GA	243	9.8%	174	14.6%
Substitution of an N by GG	51	2.1%	42	3.5%
Inversion of two adjacent residues	222	9.0%	103	8.7%
Subsitution of an end residue	1002	40.5%	421	35.4%
Subsitution of an internal residue	98	4.0%	59	5.0%
Other	861	25.8%	391	32.9%

Total	2477	100.0%	1190	100.0%

An interesting common type of *de novo *sequencing error is the inversion of two adjacent residues (Table 3). An example of this error type is a spectrum with the sequence tag of **JQ***JYR *and the Sequest identification of R.FWTD**QI***LYR*L.-. The sequence tag would match the Sequest identification by inversing the two residues Q and J in bold type. Inversions can involve any combination of amino acids. The relative order of two adjacent residues in a sequence tag is determined from fragment ions between them. It is difficult to explain why two adjacent residues can be mistakenly inversed so frequently. Note that, even if two adjacent residues in a peptide are indeed inversed from their order in the sequence database, Sequest would still likely identify this peptide as the best match for the spectrum and report the peptide sequence in the sequence database.

A much larger percentage of incorrect sequence tags have a single wrong end residue than have a single wrong internal residue (Table 3). This position-dependent bias arises from Vonode's tendency to extend a sequence tag to the longest possible. This improves average tag length, but makes the end residues far more likely to be wrong than internal residues. In light of this, it is recommended that, when matching a sequence tag to a sequence database, a mismatch at an end residue of the sequence tag should be considered as a probable *de novo *sequencing error.

Together, these five types of minor errors account for 74% of incorrect top sequence tags and 65.5% of incorrect consensus sequence tags. If all the intersection spectra are considered, 84% of the consensus sequence tags match Sequest identifications exactly, 10.5% of them mismatch due to one of these minor errors, and only 5.5% of them mismatch due to other types of errors. Similarly, there are only 5.4% of top sequence tags from the intersection spectra that mismatch Sequest identifications due to other types of errors. Therefore, *de novo *sequencing results should be used with consideration of these common types of minor errors.

## Conclusion

In this study, a *de novo *sequencing algorithm, Vonode, was developed specifically for high-resolution MS/MS data. Vonode has a unique scoring system that takes advantage of the excellent mass accuracy, sensitivity, and dynamic range of Orbitrap and FT-ICR mass analyzers. The *de novo *sequencing performance of Vonode was benchmarked in terms of accuracy, average sequence tag length, and total number of sequenced spectra using a 24-hour shotgun proteomics measurement of *R. palustris*. Using the new consensus sequence tag approach, 11,422 sequence tags with an average length of 5.5 were inferred at 84% accuracy from a total of 40,297 input spectra. This represents a significant improvement from the established PepNovo v2.0 algorithm for analyzing high-resolution MS/MS data.

Obtaining sequence tags is the first step towards identification of post-translational modifications and amino acid polymorphisms that may be missed by database searching algorithms [31,32]. In future work, a separate algorithm will be developed to use sequence tags to search a protein sequence database and reconstruct many candidate peptides by considering a large number of chemical modifications [33,34] or polymorphisms [35,36]. To find the most likely modifications or polymorphisms, the candidate peptides will be evaluated by matching their theoretical spectra against the measured spectrum, which is analogous to the scoring strategy commonly used by database searching algorithms. In this two-step approach, a *de novo *sequencing algorithm provides short sequence tags to constraint database searches for modifications or polymorphisms and a database-searching-like algorithm pinpoints optimum identifications. We showed here that the Vonode algorithm provides reliable *de novo *sequencing results from high-resolution MS/MS data.

## Authors' contributions

CP and NFS developed the Vonode algorithm. BHP designed the optimum path finding method. WHM and PAC developed the SQAMA program. NCV, JFB and RLH designed the study and acquired experimental data. All authors read and approved the final manuscript for publication.

## Supplementary Material

Additional file 1 **Figure S1, Distribution of relative mass errors in an LTQ MS/MS dataset**. Comparison of distributions of relative mass errors indicates that Orbitrap MS/MS (Figure 3) provides a much higher mass accuracy for *de novo *sequencing than LTQ MS/MS. Click here for file

Additional file 2 Table S1, Top sequence tags inferred by Vonode and their verification using Sequest identification results. Click here for file

Additional file 3 **Figure S2, *De novo *sequencing performance comparison of Vonode and PepNovo for peptides at different charge states**. The performance is analyzed for Vonode top sequence tags (A), PepNovo top sequence tags (B), Vonode consensus sequence tags (C), and PepNovo consensus sequence tags (D) from three different charge states (the blue curve for +1 peptides, the green curve for +2 peptides, and the red curve for +3 and higher charge state peptide). The thresholds are 1, 2, ..., 15 for Vonode and 0.50, 0.55, ..., 1.00 for PepNovo. The performance at each threshold is defined by the number of sequence spectra (x-axis), the accuracy (y-axis), and the average tag length (text labels of the data points). Click here for file

Additional file 4 **Figure S3, *De novo *sequencing performance comparison of Vonode and PepNovo for sequence tags at different mass coverages**. The performance is analyzed for Vonode top sequence tags (A), PepNovo top sequence tags (B), Vonode consensus sequence tags (C), and PepNovo consensus sequence tags (C) from three ranges of mass coverages (the blue curve for 0% ~20%, the green curve for 20% ~40%, and the red curve for 40% and above). The thresholds are 1, 2, ..., 15 for Vonode and 0.50, 0.55, ..., 1.00 for PepNovo. The performance at each threshold is defined by the number of sequence spectra (x-axis), the accuracy (y-axis), and the average tag length (text labels of the data points). Click here for file

Additional file 5 Table S2, Consensus sequence tags inferred by Vonode and their verification using Sequest identification results. Click here for file
