# Supplementary figures and images for: A high-throughput de novo sequencing approach for shotgun proteomics using high-resolution tandem mass spectrometry
Source: BMC Bioinformatics. 2010 Mar 5;11:118. doi: 10.1186/1471-2105-11-118 (PMC2838866; doi:10.1186/1471-2105-11-118)

Figure S1.

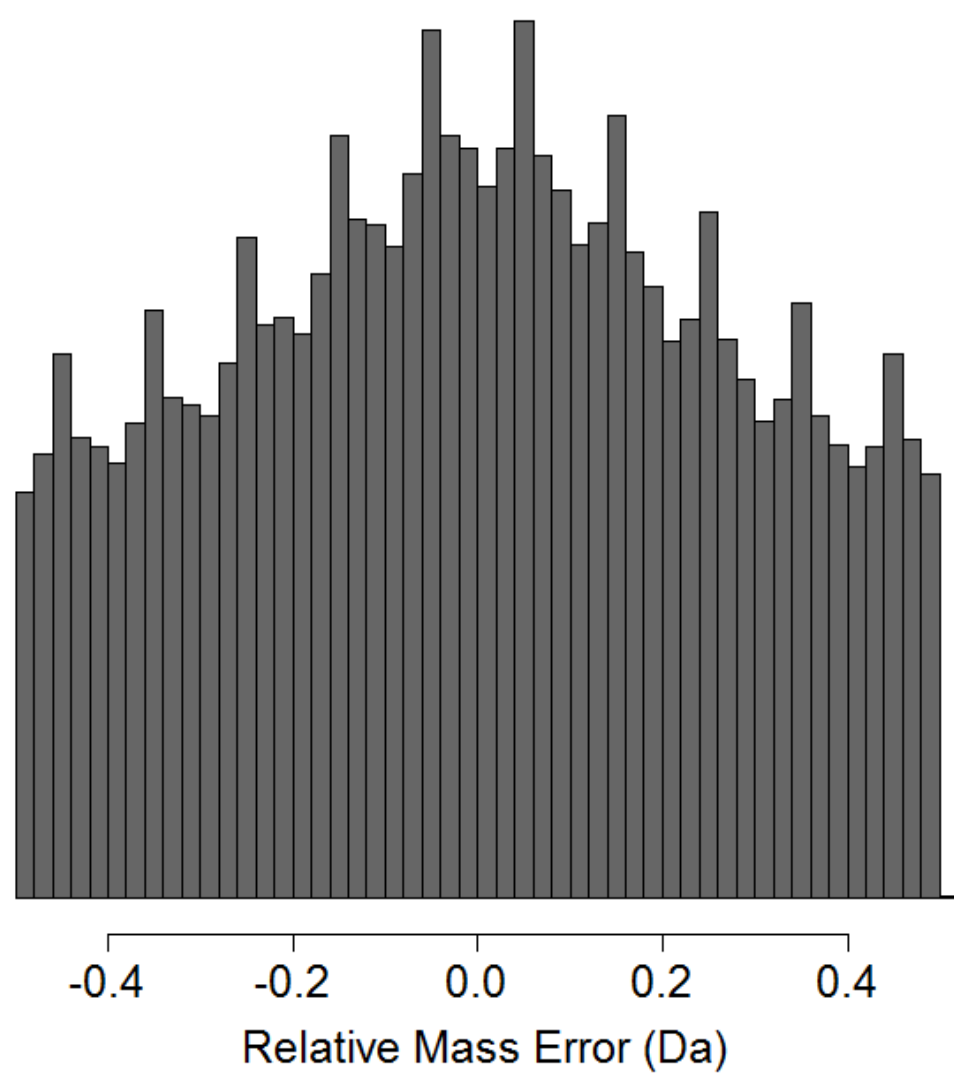

Supplement: Additional file 1 — Figure S1, Distribution of relative mass errors in an LTQ MS/MS dataset. Comparison of distributions of relative mass errors indicates that Orbitrap MS/MS (Figure 3) provides a much higher mass accuracy for de novo sequencing than LTQ MS/MS. [file 1471-2105-11-118-S1.PDF]

Figure S2.

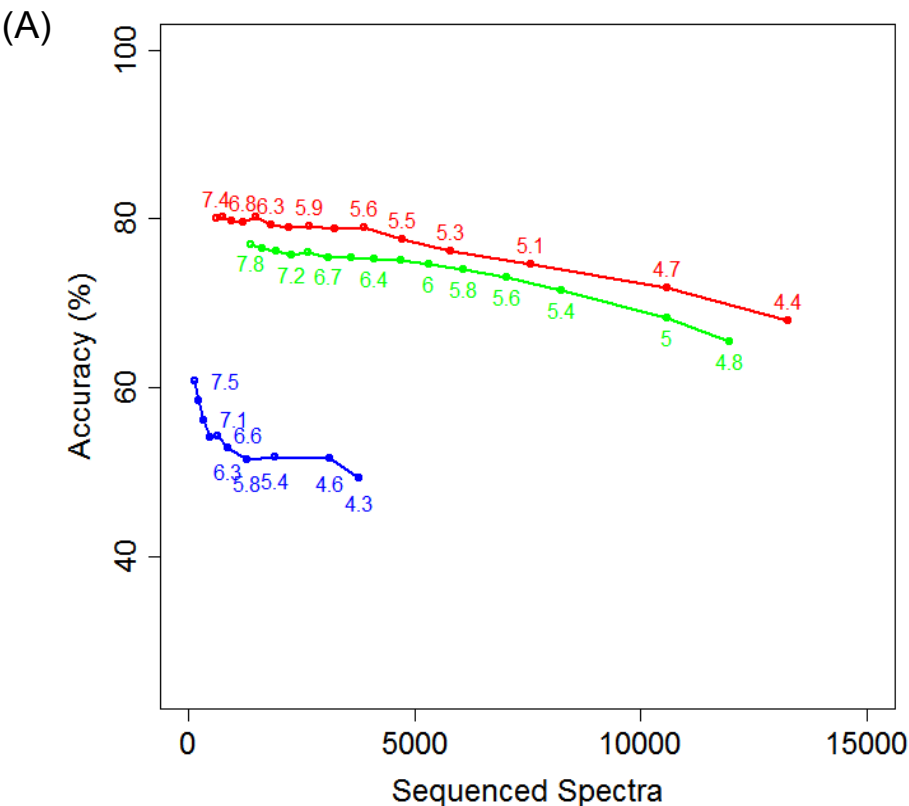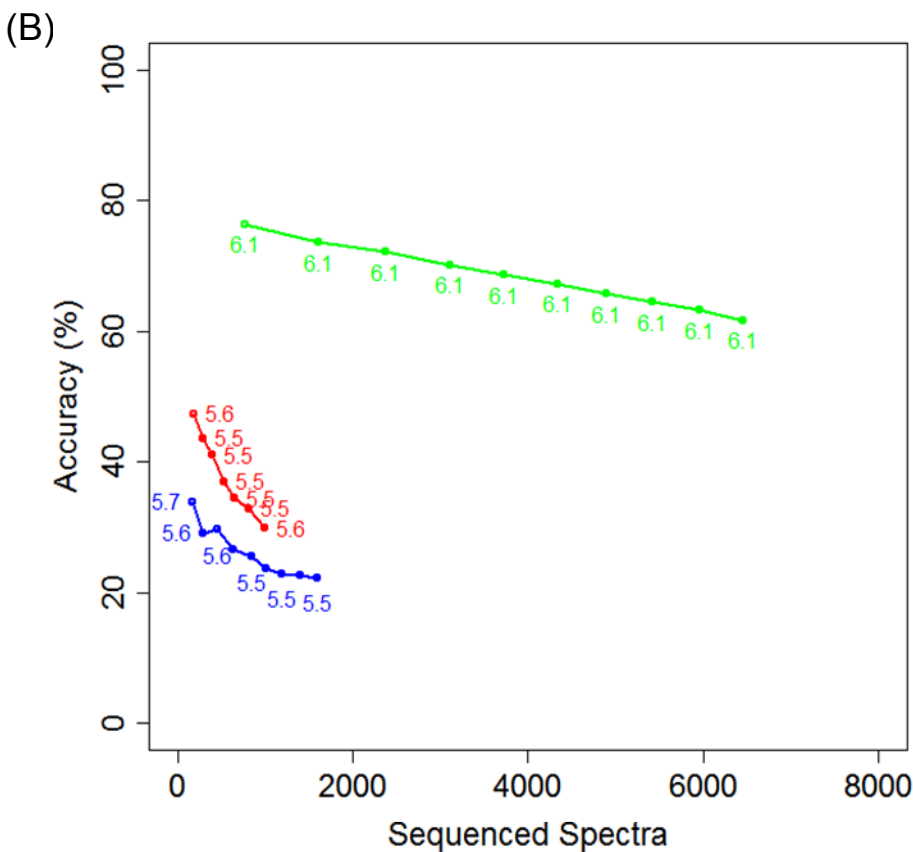

Figure S2. (Continued)

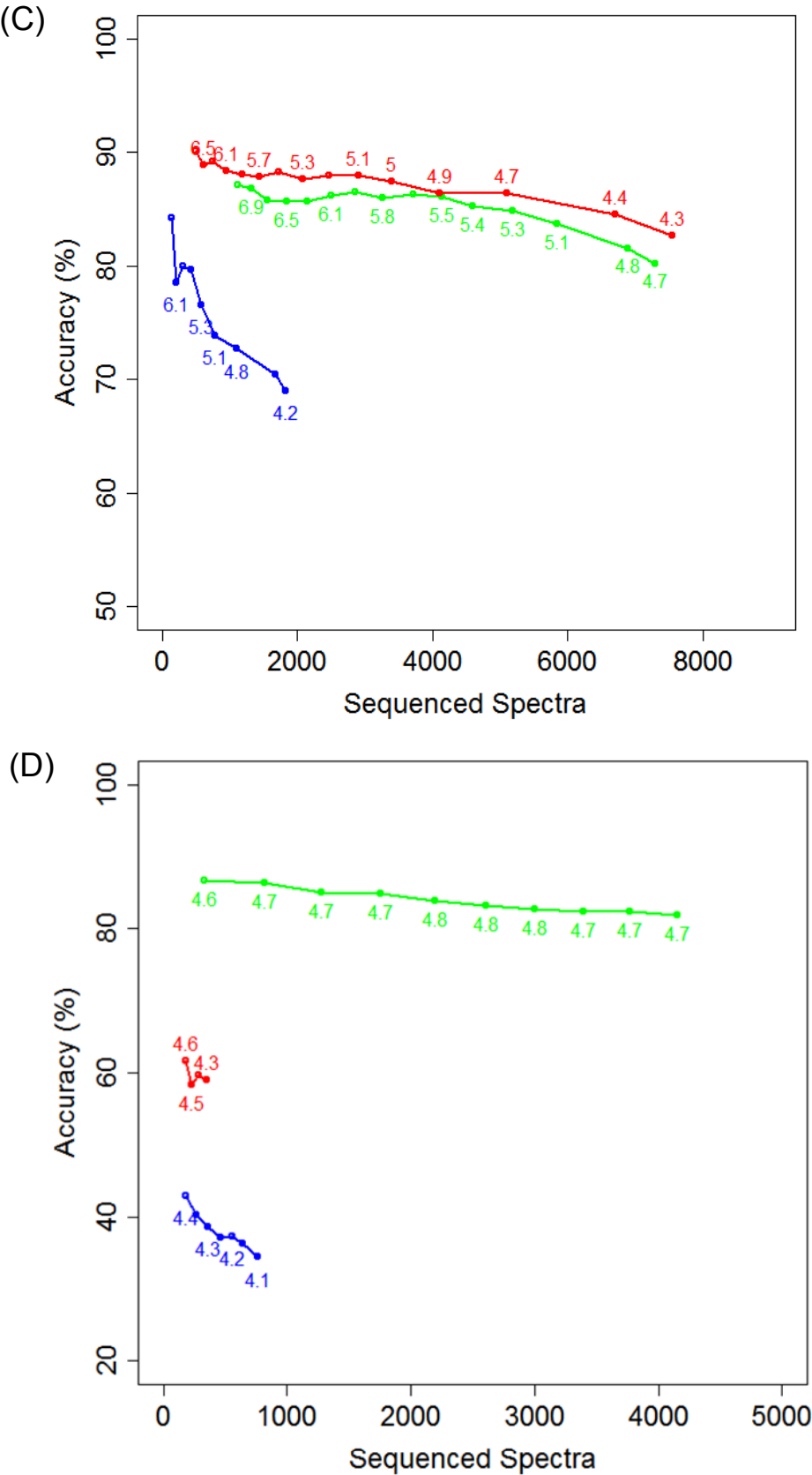

Supplement: Additional file 3 — Figure S2, De novo sequencing performance comparison of Vonode and PepNovo for peptides at different charge states. The performance is analyzed for Vonode top sequence tags (A), PepNovo top sequence tags (B), Vonode consensus sequence tags (C), and PepNovo consensus sequence tags (D) from three different charge states (the blue curve for +1 peptides, the green curve for +2 peptides, and the red curve for +3 and higher charge state peptide). The thresholds are 1, 2, ..., 15 for Vonode and 0.50, 0.55, ..., 1.00 for PepNovo. The performance at each threshold is defined by the number of sequence spectra (x-axis), the accuracy (y-axis), and the average tag length (text labels of the data points). [file 1471-2105-11-118-S3.PDF]

Figure S3.

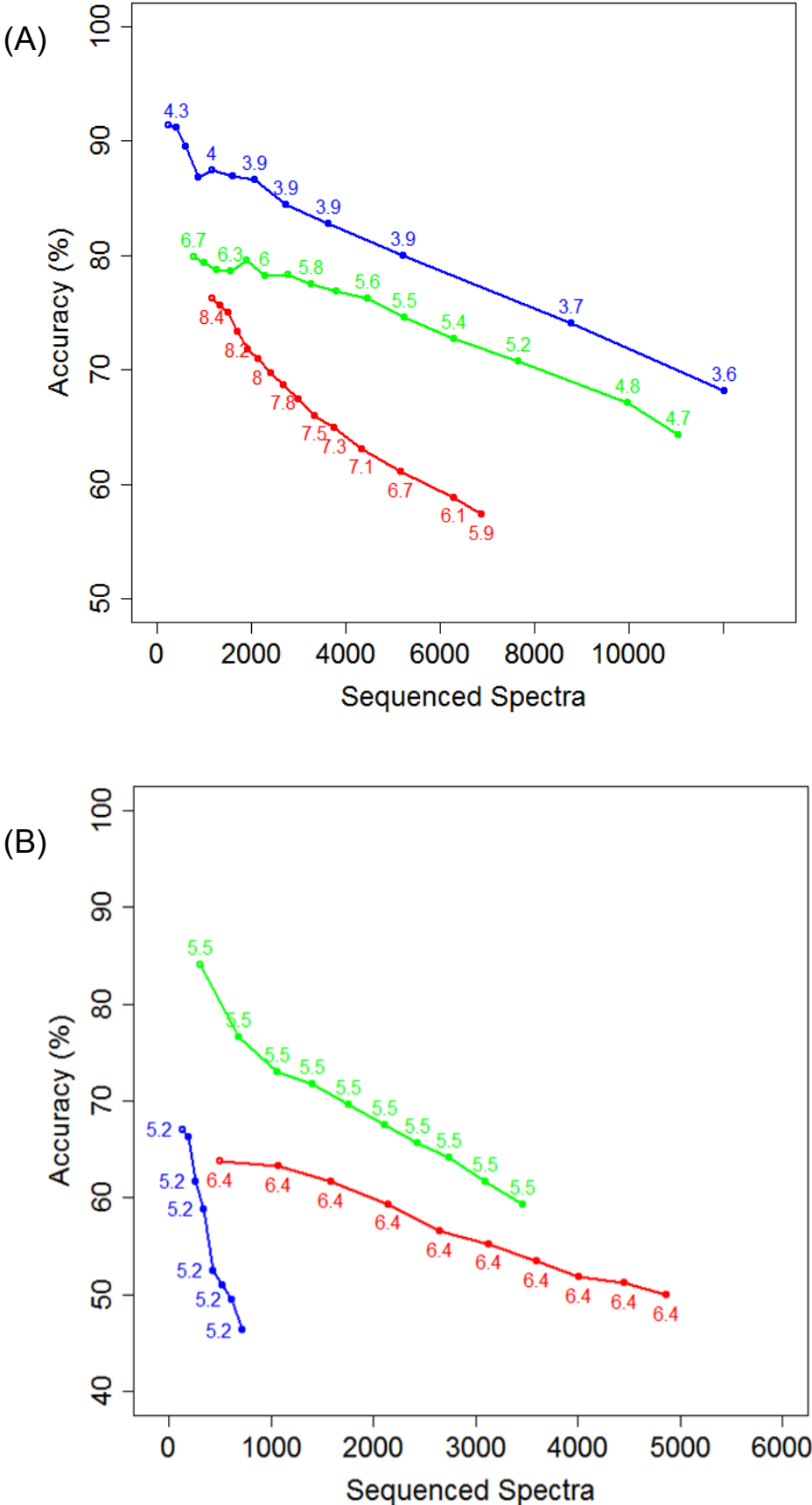

Figure S3. (Continued)

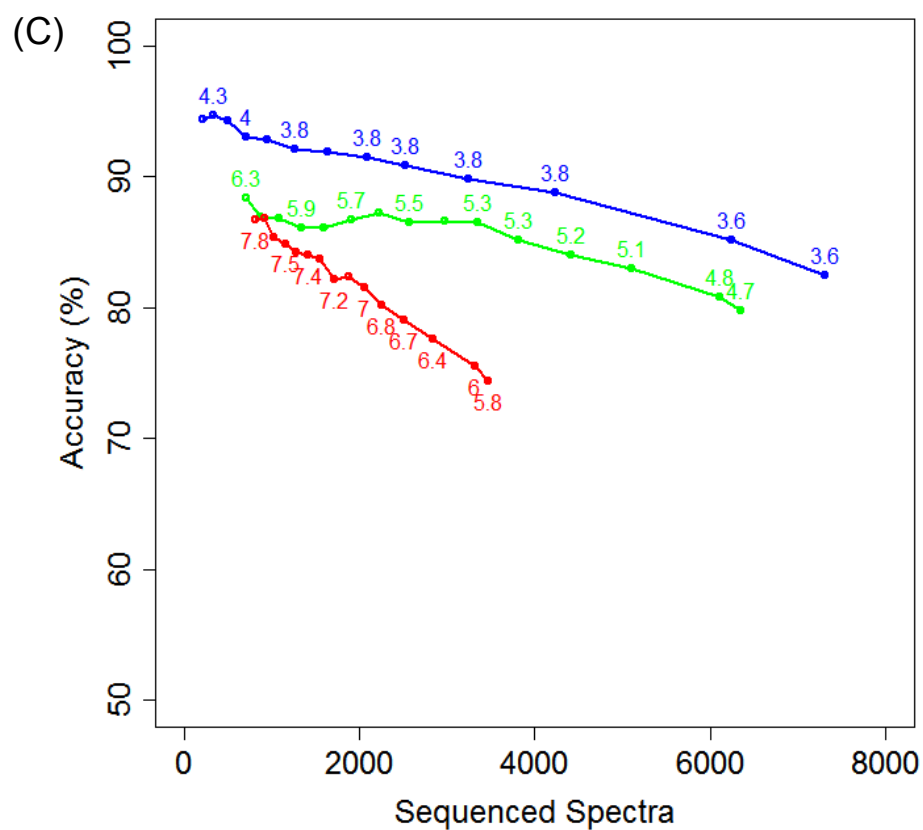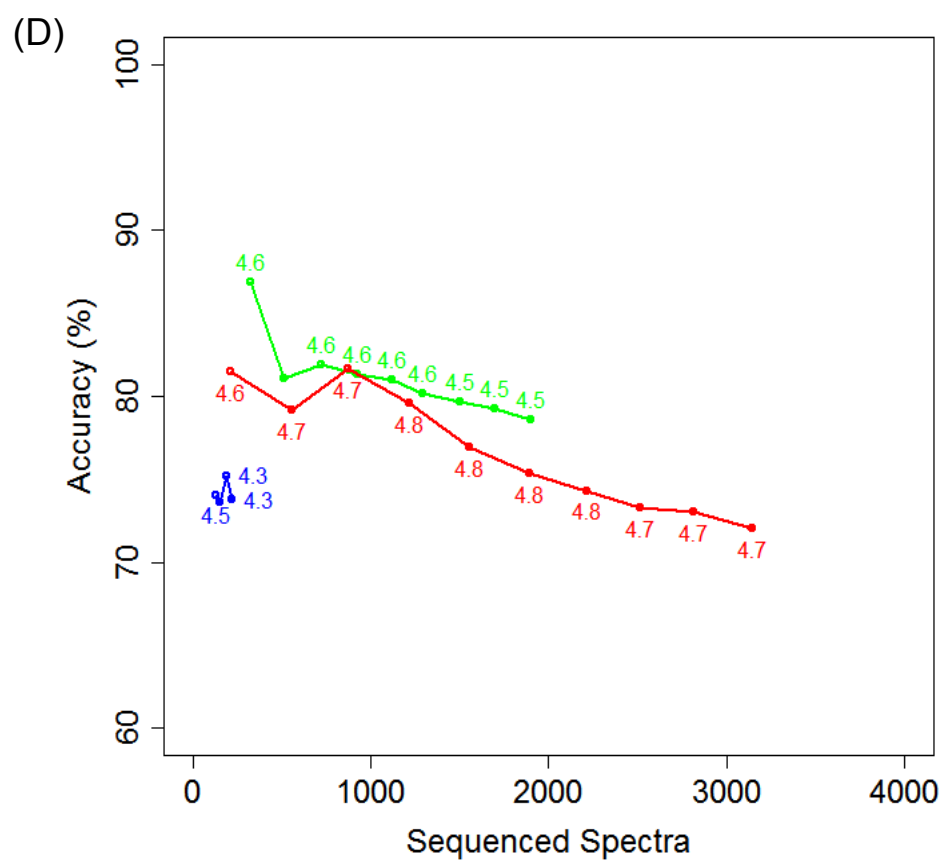

Supplement: Additional file 4 — Figure S3, De novo sequencing performance comparison of Vonode and PepNovo for sequence tags at different mass coverages. The performance is analyzed for Vonode top sequence tags (A), PepNovo top sequence tags (B), Vonode consensus sequence tags (C), and PepNovo consensus sequence tags (C) from three ranges of mass coverages (the blue curve for 0% ~20%, the green curve for 20% ~40%, and the red curve for 40% and above). The thresholds are 1, 2, ..., 15 for Vonode and 0.50, 0.55, ..., 1.00 for PepNovo. The performance at each threshold is defined by the number of sequence spectra (x-axis), the accuracy (y-axis), and the average tag length (text labels of the data points). [file 1471-2105-11-118-S4.PDF]
